# MEGADOCK 3.0: a high-performance protein-protein interaction prediction software using hybrid parallel computing for petascale supercomputing environments

**DOI:** 10.1186/1751-0473-8-18

**Published:** 2013-09-03

**Authors:** Yuri Matsuzaki, Nobuyuki Uchikoga, Masahito Ohue, Takehiro Shimoda, Toshiyuki Sato, Takashi Ishida, Yutaka Akiyama

**Affiliations:** 1 Graduate School of Information Science and Engineering, Tokyo Institute of Technology, 2-12-1 Ookayama, Meguro-ku, Tokyo 152-8550, Japan; 2 Department of Physics, Chuo University, 1-13-27 Kasuga, Bunkyo-ku, Tokyo 112-8551, Japan; 3 Grand Challenge Applications Project for Life Sciences, Next-Generation Integrated Simulation of Living Matter, Computational Science Research Program, Riken, 2-1 Hirosawa, Wako, Saitama 351-0198, Japan; 4 Education Academy of Computational Life Sciences, Tokyo Institute of Technology, 2-12-1 Ookayama, Meguro-ku, Tokyo 152-8550, Japan; 5 Research Fellow of the Japan Society for the Promotion of Science, 8 Ichibancho, Chiyoda-ku, Tokyo 102-8472, Japan; 6 Mizuho Information & Research Institute, Inc., 2–3 Kanda-Nishikicho, Chiyoda-ku, Tokyo 101-8443, Japan

**Keywords:** Parallel computing, Protein docking, Protein-protein interaction network, Protein-protein interaction prediction

## Abstract

**Background:**

Protein-protein interaction (PPI) plays a core role in cellular functions. Massively parallel supercomputing systems have been actively developed over the past few years, which enable large-scale biological problems to be solved, such as PPI network prediction based on tertiary structures.

**Results:**

We have developed a high throughput and ultra-fast PPI prediction system based on rigid docking, “MEGADOCK”, by employing a hybrid parallelization (MPI/OpenMP) technique assuming usages on massively parallel supercomputing systems. MEGADOCK displays significantly faster processing speed in the rigid-body docking process that leads to full utilization of protein tertiary structural data for large-scale and network-level problems in systems biology. Moreover, the system was scalable as shown by measurements carried out on two supercomputing environments. We then conducted prediction of biological PPI networks using the post-docking analysis.

**Conclusions:**

We present a new protein-protein docking engine aimed at exhaustive docking of mega-order numbers of protein pairs. The system was shown to be scalable by running on thousands of nodes. The software package is available at: http://www.bi.cs.titech.ac.jp/megadock/k/.

## Background

Living cells are maintained by a multitude of molecular interactions. The regulatory interactions among the thousands of proteins (Protein-Protein Interaction, PPI) in a human cell are currently being elucidated. A detailed knowledge of such interactions will be crucial for understanding the mechanisms that underlie diseases and for the development of a new generation of drugs.

The next challenge is to perform interactome level large-scale analysis by fully utilizing protein tertiary structures. In order to address this problem we proposed a large-scale PPI prediction method based on exhaustive protein docking and post-docking analysis [1-3]. Using this system, we input protein tertiary structure data to acquire predictions of possible interacting pairs.

For example, when reconstructing the human apoptosis pathway [4], 158×158 potential combinations of structures were considered. We have already applied our system to 44×44 (subset of Protein-Protein Docking Benchmark 2.0 [5]) and 89×89 (structures of bacterial chemotaxis proteins) scale analyses [1,6]. In real biology problems, such as searching for the drug induced pathway of EGFR (Epidermal Growth Factor Receptor) signaling, about 200 proteins need to be examined. In our preliminary survey on the EGFR pathway and related proteins data, we identified about 2000 structures corresponding to these proteins. Therefore, the PPI network prediction system needs to handle about 2000×2000 combinations of protein structures.

To solve such large-scale problems, a highly efficient computing system is necessary. High performance computers are currently being developed and built [7]. Some top ranked supercomputers have shown a peak performance of 27 petaflops (Titan, Oak Ridge National Laboratory, USA) and 11 petaflops (K computer, RIKEN, Advanced Institute of Computer Science (AICS), Japan) in November 2012.

We have implemented a protein docking system “MEGADOCK” suitable for running on supercomputers by using hybrid parallelization (MPI, Messeage Passing Interface/OpenMP, Open Multi-Processing), where a number of docking processes are distributed among the nodes by MPI with each docking process also calculated in parallel by threads by OpenMP within one node. Data parallelization showed almost linear scaling up to 24,576 nodes on K computer (RIKEN AICS, Japan). In addition, we also designed MEGADOCK using a simple score model to reduce the number of calculations required for protein docking.

We expect the proposed method can be a useful tool in bioinformatics and systems biology area as a basic tool, assuming we can utilize ~10,000 CPUs.

### Implementation

We adopted two strategies to make MEGADOCK suitable for large-scale docking simulations performed on supercomputers. First we devised a novel score function with real Pairwise Shape Complementarity, rPSC that enables us to calculate two aspects of interactions using one convolution function with Fast Fourier Transform (FFT) calculations. This setup markedly reduces the time required for each docking calculation per protein pair. Second, we implemented MEGADOCK by hybrid parallelization (MPI/OpenMP) in order to conduct large numbers of docking jobs for PPI network predictions.

**Figure 1:**
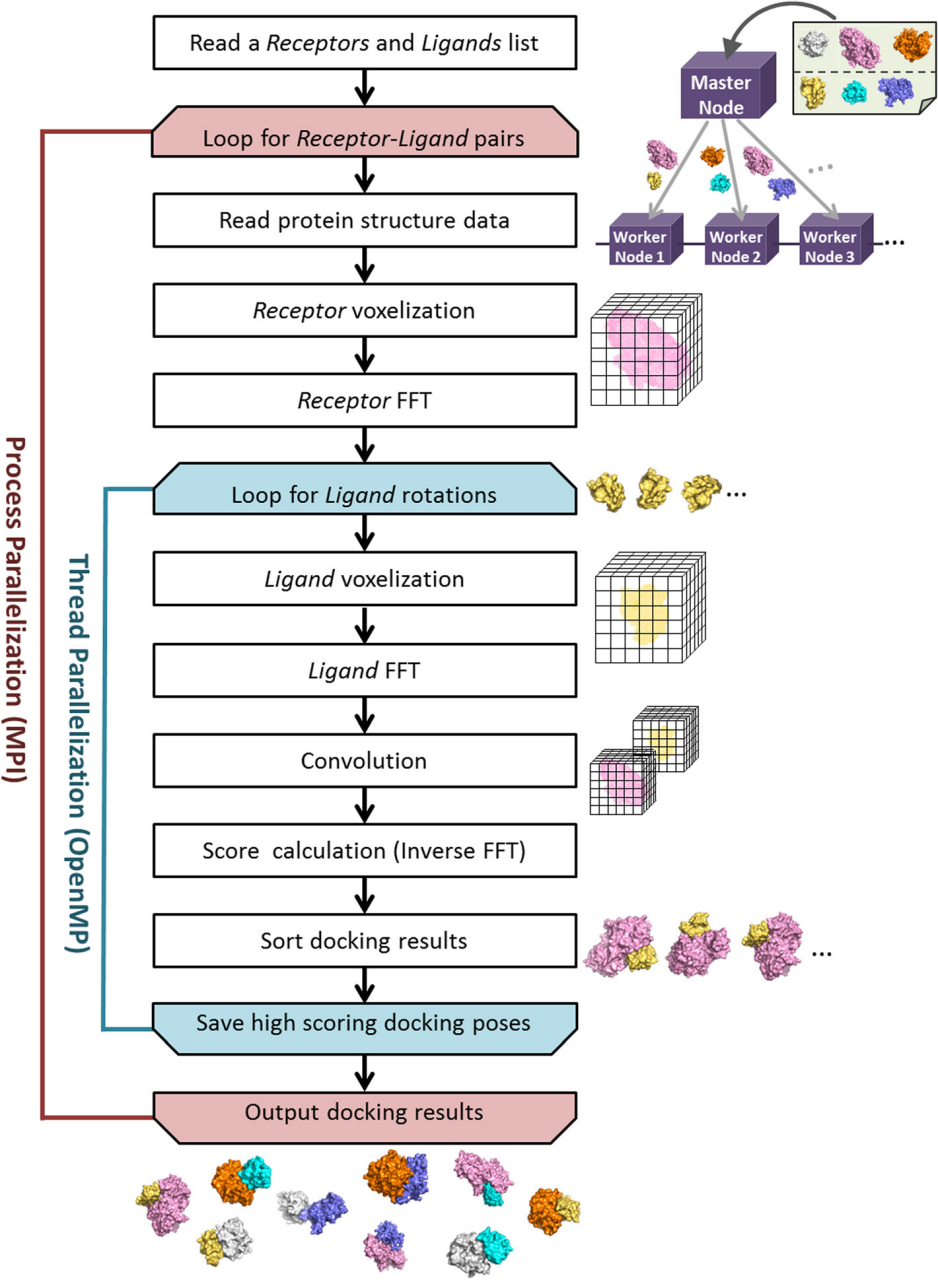
**Flow chart of the MEGADOCK docking process.** A master node gets a list of docking targets and distributes each job to the available nodes. Each node calculates one docking job by thread parallelization.

The overall procedure of MEGADOCK is shown in Figure 1. On the cluster computers, a master node gets a list of protein structures and distributes the docking jobs to available nodes. Upon docking of two proteins (here we call them “receptor” and “ligand”, apart from biological definition, to indicate two docked proteins), a “ligand” is rotated to various orientations and translated in the space around the “receptor”, which is fixed during docking calculation, to search for the best scoring positions. These processes are parallelized by threads.

### Docking score function

MEGADOCK includes a rigid-body docking system that searches relevant interacting poses between the target protein tertiary structures. The rigid-body docking process is mainly based on shape complementarity of the target proteins without considering conformational changes upon formation of the complex structures.

Docking scores are defined by an equation consisting of the shape complementarity term *G* and the electrostatic interaction term *E*. The target protein pairs R (receptor) and L (ligand) are first allocated on the 3-D voxel space **V,** 1.2Å × 1.2Å × 1.2Å. Then the voxel values are assigned to each voxel (*l*, *m*, *n*) ∈ **V** according to the location in a protein, open space and core. We use our original scoring function called “real Pairwise Shape Complementarity” (rPSC) for the shape complementarity term *G* as follows [2]:

$$G_{R}\left(l,m,n\right)=\left\{\begin{array}{ll} \#\mathrm{of}\;R\;\mathrm{atoms}\;\mathrm{within}\;(3.6Å+r_{\mathrm{vdw}}\;\mathrm{of}\;R\;\mathrm{atoms}\;\mathrm{in}\;\mathrm{the}\;\mathrm{voxel}\;\left(l,m,n\right)) & \;\left(\mathrm{open}\;\mathrm{space}\right)\\ -45 & \;\left(\mathrm{core}\;\mathrm{of}\;R\right) \end{array}\right)$$

$$G_{L}\left(l,m,n\right)=\left\{\begin{array}{cc} 0 & \left(\mathrm{open}\;\mathrm{space}\right)\\ 1 & \left(\mathrm{core}\;\mathrm{of}\;L\right) \end{array}\right)$$

Here *r*_*vdw*_ represents the van der Waals radius of an atom, and (*α*, *β*, *γ*) is a vector of the ligand translation.

**Figure 2:**
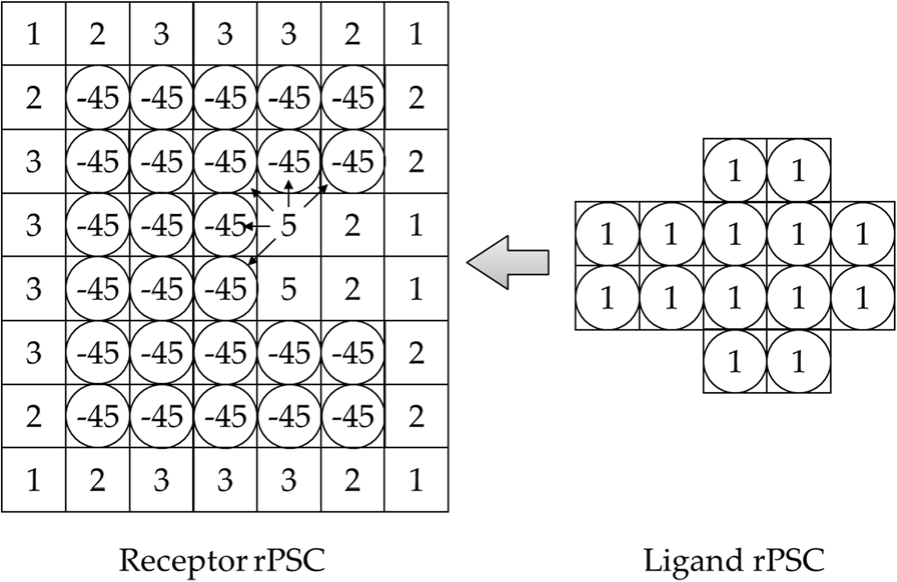
**Real pair-wise shape complementarity score model.** For each potential docking process, the sums of the overlapped voxel values are assigned as the docking score.

The advantage of rPSC is that it is a real number representation (Figure 2), thus we can put a physicochemical parameter into the imaginary part of the voxel score, which are complex numbers. Hence, it is possible to calculate docking scores by assigning only one complex number for each voxel.

For physicochemical parameters, we use the electrostatic interaction of each amino acid, *E*_*R*_(*l*, *m*, *n*) and *E*_*L*_(*l*, *m*, *n*). These values are calculated using the interaction energy in each voxel, *q* (*l*, *m*, *n*) using CHARMM19 [8] for the electrostatic charge of each atom.

Considering these terms, the overall docking score *S* is calculated as follows:

$$\begin{array}{l} R\left(l,m,n\right)=G_{R}(l,m,n)+iE_{R}(l,m,n)\\ L\left(l,m,n\right)=G_{L}(l,m,n)+\mathrm{iw}E_{L}(l,m,n)\\ S\left(\alpha ,\beta ,\gamma \right)=R\left[\sum_{l=1}^{N}\sum_{m=1}^{N}\sum_{n=1}^{N}R\left(l,m,n\right)L\left(l+\alpha ,m+\beta ,n+\gamma \right)\right] \end{array}$$

For direct execution of a simple convolution sum, *O*(*N*^6^) calculations are required. The calculation order using fast Fourier transform (FFT) algorithms, both for a discrete Fourier transform (DFT) and inverse discrete Fourier transform (IFT), is reduced to *O*(*N*^3^ log *N*) [9]. The score *S* for FFT is:

$$S\left(\alpha ,\beta ,\gamma \right)=R\left[\mathrm{IFT}\left[\mathrm{DFT}{\left[R\left(l,m,n\right)\right]}^{*}\mathrm{DFT}\left[L\left(l,m,n\right)\right]\right]\right]$$

By reducing the number of required DFT/IFT operations with rPSC, MEGADOCK in theory should perform the docking process faster than the well-known protein-protein docking tool, ZDOCK [10,11], which uses eight complex numbers per calculation.

### Hybrid parallelization

The overall procedure of MEGADOCK is shown in Figure 1. Initially, a master node distributes docking jobs to available nodes after obtaining a list of protein pairs. We parallelized the calculation of each docking processes using MPI library (Figure 1, red colored loop). After one loop of this MPI, we obtain high scoring poses among all the rotation and translation patterns of assigned protein pairs.

Each docking process in each node is parallelized to threads by OpenMP (Figure 1, blue colored loop). Upon docking, the coordinates of the ligand are repeatedly rotated and translated to search for a better complex form with the receptor. The calculations of FFT and inverse FFT for each rotation angle are performed independently. Thus, using OpenMP loop we calculate high scoring poses for various rotation angles in parallel.

The implementation is designed to run efficiently on K computer which has 88,128 nodes with 8 cores per node (i.e., total of 705,024 cores). Each node is equipped with 16 GB of memory. Flat MPI is often used for parallel applications. However, using flat MPI on numerous core systems like K computer may result in a large overhead due to handling data communication of ~700,000 cores. Thus, hybrid parallelization is efficient on such high performance computing systems.

Reducing usage of memory space is important with systems that have many cores per node and relatively small memory size. In flat MPI, the docking job of each protein pair is assigned to each core. Thus, each core requires memory space for input/output data. If a node has *n* cores, the memory space in the node should be large enough to keep data for *n* pairs of proteins (in the case of K computer, *n* = 8). In contrast, by implementing hybrid parallelization, we assign one protein pair to each node and then distribute the calculations of ligand rotation by thread parallelization. As such, each node will keep data of one pair of proteins on the memory and threads will share the input/output data on the memory. The memory size needed for docking is dependent on protein size. This implementation is feasible when considering calculations of large proteins. Thus, we implemented MEGADOCK by hybrid parallelization.

## Results and discussion

### Dataset

We used a general benchmark dataset for protein docking (Protein-Protein Docking Benchmark 4.0, [12]) to evaluate the accuracy of predicted docking poses generated by MEGADOCK. This benchmark set comprises 176 known complexes and included both a “bound” and “unbound” set. The “bound” set is composed of protein structures prepared by separating individual proteins from the crystal structures of 176 protein complexes. The “unbound” data means that each protein structure is taken from the isolated form of crystals rather than complex form. The “unbound” dataset includes protein structural data corresponding to the same set of proteins in the “bound” dataset. Structural differences in bound and unbound form in RMSD are shown in the reference [12].

**Figure 3:**
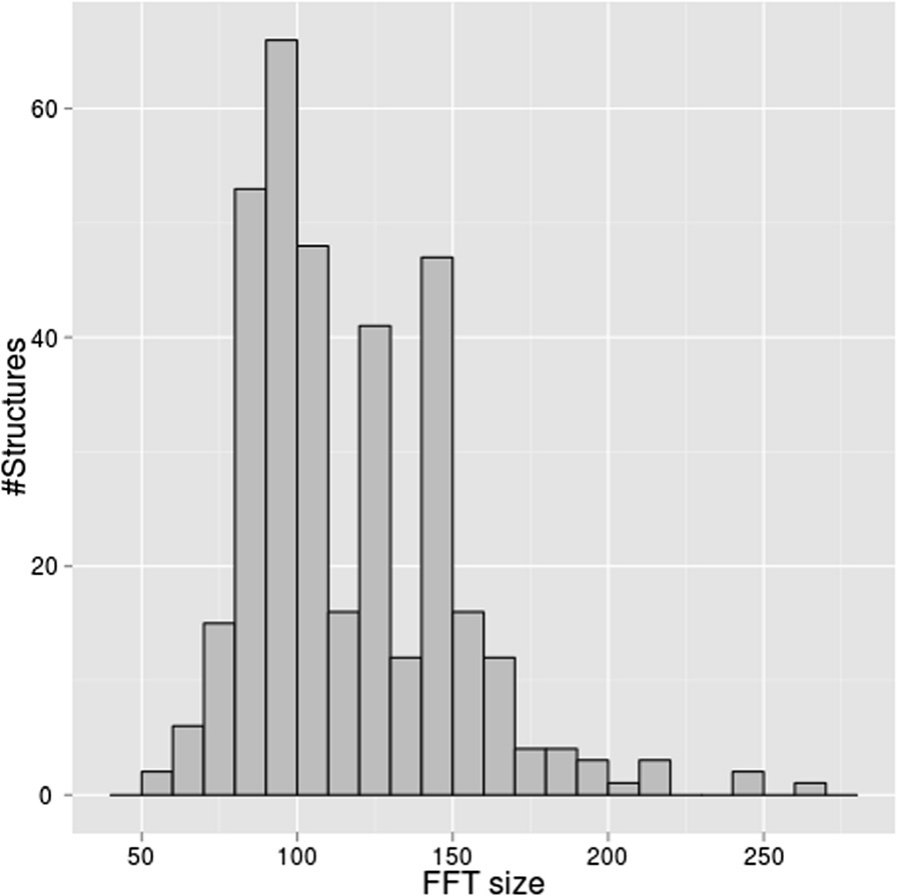
FFT size of various proteins in protein-protein docking benchmark 4.0 (176 Protein complexes, 352 structures).

We also evaluated elapsed time for docking calculations over this dataset (calculation of 176 protein complex predictions). Distribution of size of FFT for protein in this dataset is shown in Figure 3. Time consumed for FFT is expected to be longer with larger size of FFT.

For measuring thread parallel scalability, we conducted dockings of a protein complex from PDB, 1ACB (chain E and I). The size of FFT is 108 in this case. Parallel scalability over nodes using MPI was measured by conducting exhaustive docking of 220 different proteins (220×220 dockings), with an FFT size of 140.

### Test environment

Parallel scalability of MEGADOCK was measured on two supercomputing environments, TSUBAME (Tokyo Institute of Technology Global Scientific Information and Computing Center (GSIC), Japan) and K computer (RIKEN AICS, Japan). The most abundant node type of TSUBAME had an Intel Xeon 5670, 2.93 GHz processor, 12 cores. Each node is capable of up to 24 threads of computation by using the hyper threading technique. K computer has Fujitsu SPARC64 VIIIfx CPUs, 2 GHz, 8 cores.

### Calculation speedup

Table 1 shows the total calculation time needed for docking 176 protein complexes in Protein-Protein Docking Benchmark 4.0 [12]. MEGADOCK calculation speed was measured from the calculation time using one node and one thread to show the baseline. The measurement was conducted on a node of the TSUBAME supercomputing system (Tokyo Institute of Technology, Japan), equipped with an Intel Xeon 5670, 2.93 GHz processor. In Table 1, we list the other two FFT-based docking software packages, ZDOCK 3.0 and ZDOCK 2.3. Both versions of ZDOCK use the same FFT library [13] as MEGADOCK. Ligand FFT, inverse FFT consists large part (on average 85.1% for 176 dockings) of the calculation time as shown in Table 2. As all three software listed in Table 1 uses the same FFT library (FFTW), Table 1 shows the obtained speedup by adopting the simplified score function with rPSC compared to ZDOCK 3.0.

**Table 1:** **Total calculation time on one node and one thread to compute 176 dockings of protein-protein docking benchmark 4.0**[12]

**Name**	**Running time [hr]**	**Speedup from ZDOCK 3.0**
MEGADOCK 3.0	41.7	8.77
ZDOCK 2.3	157.3	2.32
ZDOCK 3.0	365.6	1.0

Note that the real application of the docking is expected to be run using multi-nodes on a multi-thread system. Consequently, the calculations are performed much faster than the running time given in this table.

**Table 2:** **Ratio of time spent for each process in the total docking time (Average of 176 dockings of protein-protein docking benchmark 4.0**[12]**, calculated with single thread setting)**

**Calculation**	**Ratio of time spent for the process [%] (mean±sd)**
Receptor voxelization and FFT	1.19±0.62
Ligand rotation and voxelization	6.41±3.13
Ligand FFT	40.38±2.79
Inverse FFT	45.99±2.25
Sort docking results	6.02±1.46

The dataset includes proteins of various size (Figure 3). The time required for each docking calculation is dependent on protein size. For example, a protein that requires size 120 FFT calculations (1E96) gave a calculation time of about 547 seconds. Smaller sized protein pairs, such as size 80 FFT (1GCQ) were calculated in about 155 seconds. This variation in calculation time reflects the difference of FFT calculation (size 120×120×120 and 80×80×80). The smaller protein pair (size 80 FFT) takes about 0.28 times the elapsed time compared to the larger protein pair (size 120 FFT). This ratio of elapsed time is reasonable. In theory FFT takes the order of *O*(*N*^3^ log *N*) for calculation. Therefore calculations involving a size of 80 FFT should take ~0.27 times ((80^3^ log 80)/(120^3^ log 120)) the elapsed time of a corresponding calculation involving a size of 120 FFT, which is almost the same scale as the calculation time we measured on TSUBAME.

### Parallel scalability

**Figure 4:**
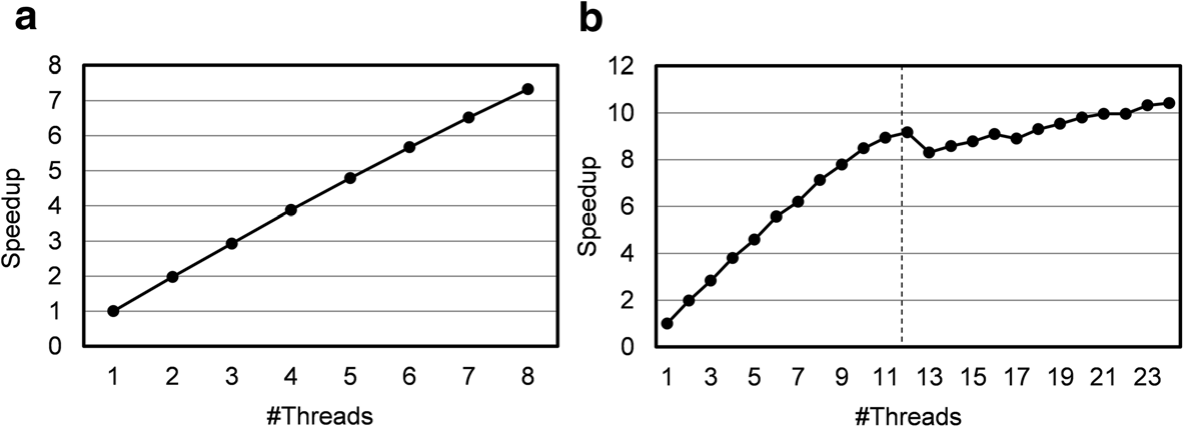
**Scalability of thread parallelization using OpenMP on (a) K computer (8 cores/node) and (b) TSUBAME (12 cores/node, hyper threading enabled).** 1ACB chain E and 1ACB chain I was used for docking. Elapsed time was measured from the mean of 30 docking processes. The right area of the dashed line shows speedup by activating hyper threading.

Figure 4 shows the thread parallel scalability of MEGADOCK by parallelizing ligand rotation and FFT calculation. The calculation time is shown as an average of 10 individual docking events with an FFT size of 108 (1ACB chain E and I) from the benchmark data. We observed a 7.33-fold speedup when using the maximum number of threads, 8 threads, on K computer compared to a single thread calculation. We observed a 9.17-fold speedup for 12 threads of calculation and a 10.42-fold speedup for 24 threads of calculation compared to a single thread calculation. Note that in TSUBAME system we measured time with hyper threading activated, so number of threads more than 12 includes slight speedup including this effect.

**Figure 5:**
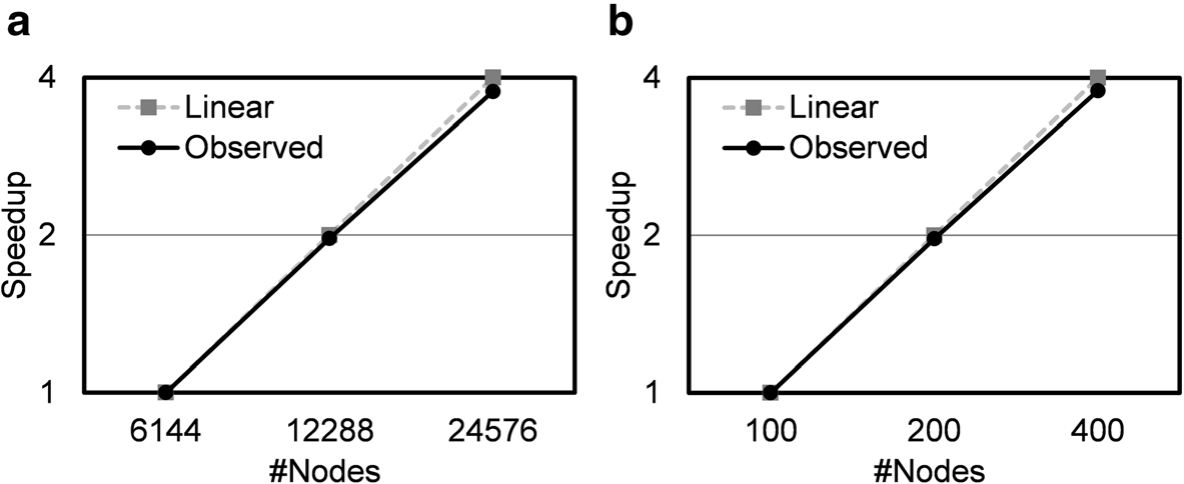
Scalability of parallelization among nodes by MPI on (a) K computer (6144 to 24576 nodes), 220×220 dockings of FFT size = 140 protein pairs; (b) TSUBAME (100 to 400 nodes), 44x44 dockings of FFT size = 140 protein pairs.

Figure 5 shows a process level parallel scalability of MEGADOCK. On K computer, where a maximum of 24,576 nodes can be used simultaneously, we measured the time needed to calculate exhaustive dockings of 220 proteins (220×220=48,000 dockings), calculated with a size of FFT 140. Calculation time using 24,576 nodes was about 3.76-fold faster than the time needed to solve the same problem on 6,144 (1/4 of 24,576 nodes) nodes. On TSUBAME, we measured the time needed to calculate exhaustive dockings of 44 proteins (44×44=1,936 dockings) using up to 400 nodes at a time. Calculation time using 400 nodes was about 3.78-fold faster than that on 100 nodes. MEGADOCK achieved almost linear scalability on both supercomputing environments.

We used a dataset of similar sized proteins (FFT size 140) for the scalability test. It is an unrealistic scenario when calculations conducted by each node are almost equal. Thus, the only possible overhead by parallelization is the job distribution and checking by the controller nodes. For real problems, which include simulating dockings of proteins with a variety of sizes, a more intelligent controller is needed to efficiently distribute docking tasks according to the protein size.

Another possible improvement to make calculation faster could be on the FFT calculation. A profiler output showed that about 86.37% of the elapsed time was used by FFT and inverse FFT calculations. Users can switch the FFT engine to similar libraries by making small changes to the MEGADOCK source code. We have tried using FFTE [14], FFTW [13] and FFT function (dvcfm1) in CSSL2 (Fujitsu Ltd., Tokyo, Japan). All three implementations yielded equivalent docking outputs. The speed of calculation differs depending on the size of the proteins. For example, CSSL2 was slightly faster (data not shown) than FFTW when applied to FFT size of 128 or other base 2 FFT calculations. By contrast, FFTW outperformed CSSL2 with docking simulations involving other sizes of protein. Thus, FFTW may be the function of choice for applications where the dataset includes proteins of various sizes.

### Application to protein-protein interaction prediction

**Figure 6:**
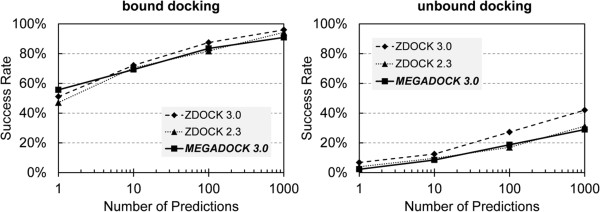
**Docking success rate of MEGADOCK.** (left) docking of benchmark 4.0 bound data, (right) docking of benchmark 4.0 unbound data.

Figure 6 shows the accuracy of protein docking by means of “Success rate”. Success rate of the top *k* high scoring docking predictions represents the percentage of protein pairs that have “correct” complex poses in the top *k* docking predictions. Here we define a prediction as being “correct” if the predicted complex has a root mean square deviation of less than 5 Å for the coordinates of the ligand (L-RMSD, Root Mean Square Deviation of the coordinates of the Ligand) to the crystal structure. L-RMSD is a measure between corresponding ligand structures when the receptor part was superimposed between two given protein complexes.

Using the decoys obtained by exhaustive docking, we conducted prediction of interacting protein pairs. The PPI network prediction method is detailed in our previous publications [1,6]. We predicted relevant interacting proteins from 44×44=1,936 combinations by docking and post-docking analysis. We obtained an F-measure value of 0.42 for this dataset. The F-measure is the harmonic mean of precision (0 to 1, where 1 means all the predicted PPIs are confirmed as correct) and recall (0 to 1, where 1 means the prediction covered all the known correct interactions).

We also evaluated our PPI prediction system by applying it to the data from the bacterial chemotaxis pathway, which is one of the typical problems in systems biology. For this dataset we obtained an F-measure value of 0.52.

## Conclusions

In this study, we implemented a high-throughput and ultra-fast PPI network prediction system “MEGADOCK” suitable for massively paralleled large-scale analysis of millions of protein combinations. The docking engine of MEGADOCK was implemented by parallelization techniques and shown to be scalable on massively parallel computing environments. MEGADOCK is ideally suited to a large-scale computing system.

Using the MEGADOCK framework, it is simple to improve score functions and test it on a large dataset. For example, incorporating desolvation effects were considered in a previous report [15] and will be included in future releases. MEGADOCK is an open-source software package, making it easy for the user to apply different score functions. A further advantage is that users can change many parameters, such as the weight of each term of score functions.

Protein docking based PPI network prediction has various applications. For example, Acuner Ozbabacan *et al.* analyzed the human apoptosis pathway using the docking based system PRISM [16]. This problem required calculation of 158×158 combinations of structures. Further useful applications may include for example the EGFR signaling pathway (where approximately 2000×2000 combinations of tertiary structures need to be examined), which is implicated in the onset of lung cancer and various other serious diseases.

### Availability and requirements

•Project name: MEGADOCK

•Project home page: http://www.bi.cs.titech.ac.jp/megadock/k/

•Operating system(s): Linux

•Programming language: C++

•Other requirements: FFTW, MPI, OpenMP

•License: GPLv3

•Any restrictions to use by non-academics: No (licence holds)

## Abbreviations

DFT: Discrete Fourier transform; EGFR: Epidermal growth factor receptor; FFT: Fast Fourier transform; IFT: Inverse discrete Fourier transform; L-RMSD: Root mean square deviation for the coordinates of the ligand; MPI: Message passing interface; OpenMP: Open multi-processing; PDB: Protein data bank; PPI: Protein-protein interaction; rPSC: Real pairwise shape complementarity.

## Competing interests

The authors declare that they have no competing interests.

## Authors’ contributions

YM, NU and MO carried out the docking experiments and measurements of parallelization effects on K computer and TSUBAME. YM carried out measurements by using different FFT engines and packaging of the software. YM, NU and MO drafted the manuscript. MO, TShimoda, TI and TSato implemented the MEGADOCK software. YA conceived of the study, and participated in its design and coordination. All authors have read and approved the final manuscript.
